# Shift of musical hallucinations to visual hallucinations after correction of the hearing deficit in a patient with Lewy body dementia: a case report

**DOI:** 10.1186/s13256-021-03039-2

**Published:** 2021-09-09

**Authors:** Alexandre Montalvo, Eryco Azevedo, Alexandre de Mendonça

**Affiliations:** 1 Faculdade de Medicina da Universidade de Lisboa, Lisbon, Portugal; 2 Faculdade de Ciências Médicas, Universidade do Estado do Rio de Janeiro, Rio de Janeiro, Brazil

**Keywords:** Hallucinations, Musical, Auditory, Sensory modality, Shift, Deafness, Dementia with Lewy bodies

## Abstract

**Background:**

Musical hallucinations are a particular type of auditory hallucination in which the patient perceives instrumental music, musical sounds, or songs. Musical hallucinations are associated with acquired hearing loss, particularly within the elderly. Under conditions of reduced auditory sensory input, perception-bearing circuits are disinhibited and perceptual traces released, implying an interaction between peripheral sensory deficits and central factors related to brain dysfunction.

**Case presentation:**

A 71-year-old Caucasian man with hearing loss complained of memory difficulties and resting tremor of the right upper limb in the previous 2 years. He already had difficulties in instrumental activities of daily life. Neurological examination showed Parkinsonian signs and hypoacusia. Neuropsychological examination identified deficits in executive functions and memory tests. Brain computerized tomography and nuclear magnetic resonance scans showed mild cortical and subcortical atrophy. The clinical diagnosis of possible dementia with Lewy bodies was established. Five years later, the patient began complaining of musical hallucinations. There had been no previous change in medication. An otorhinolaryngologist diagnosed age-related hearing loss and prescribed bilateral hearing aids. After using the hearing aids, the patient did not hear the songs any longer, only some tinnitus, described as a whistle. However, at the same time, the patient started experiencing visual hallucinations he never had before.

**Discussion:**

To our knowledge, the immediate shift of hallucinations from one sensory modality to another sensory modality when perception is improved has not been previously described. This report emphasizes the interaction between brain pathology and sensory deficits for the genesis of hallucinations, and reinforces the theory that attention and control networks must couple properly to the default mode network, as well as integrate and select adequately peripheral signals to the somatosensory cortices, in order to keep a clear state of mind.

**Conclusion:**

The clinician should bear in mind and let the patient know that improving one sensory modality to ameliorate hallucinations may sometimes paradoxically lead to hallucinations in a different sensory modality.

## Background

Musical hallucinations are a particular type of auditory hallucinations in which the patient perceives instrumental music, musical sounds, or songs. Patients may have preserved insight regarding the unreality of the phenomenon, and in this case the term musical hallucinosis should be more appropriate. Musical hallucinations have been labelled Oliver Sacks’ syndrome after the British neurologist and author of the book Musicophilia [1]. Musical hallucinations may have different causes, namely psychiatric disorder, focal brain lesion, epilepsy, and intoxication [2]. Musical hallucinations were also, for long time, known to be associated with acquired hearing loss, particularly in the elderly [3, 4]. Admittedly, under conditions of reduced auditory sensory input, perception-bearing circuits would be disinhibited and perceptual traces released. This phenomenon was proposed to be akin to the appearance of complex visual hallucinations in blind people with preservation of insight, as first described by the Swiss philosopher and naturalist Charles Bonnet in the eighteenth century [5]. Interestingly, in these early reports, the concomitance and the interaction of peripheral sensory deficits factors and central factors related to brain dysfunction were already emphasized [3, 6].

We describe the case of a patient with dementia with Lewy bodies (DLB) and age-related hearing loss in whom the musical hallucinations shifted to visual hallucinations after correction of hearing deficit with hearing aids.

## Case presentation

A 71-year-old Caucasian man complained of memory difficulties and resting tremor of the right upper limb in the previous 2 years. He had difficulty learning new tasks like using a different mobile phone. His wife took care of his medication. He still bought the newspaper and did simple shopping in the neighborhood with the aid of a shopping list, but no longer handled financial or other important matters. There were neither hallucinations nor clear clinical fluctuation at this time. Regarding personal history, the patient suffered from controlled arterial hypertension and diabetes mellitus, angina pectoris, obstructive sleep apnea, benign prostatic hyperplasia, and major depression. He also had cataract surgery in the left eye, as well as hearing loss. Neurological examination showed positive Myerson’s sign, tongue tremor, resting tremor, and cogwheel rigidity of the right arm, mild dystonic posture of the right hand, Parkinsonian gait with mild alteration of postural reflexes, and writing tremor, and confirmed the hypoacusia. Neuropsychological examination [7] identified deficits in executive functions (Trail Making Test B) as well as memory tests, namely logical memory (stories from Wechsler Memory Scale), associative learning (Word Pairs), and word list learning (California Verbal Learning Test). Other cognitive functions were maintained. The patient had mild depressive symptoms scoring 6 on the Geriatric Depression Scale (15 items) [8] and was clearly deteriorated according to the Blessed Dementia Scale score (5.5 points) [9]. Brain computerized tomography and nuclear magnetic resonance scans showed mild cortical and subcortical atrophy. The clinical diagnosis of possible dementia with Lewy bodies (DLB) [10] was established. The patient’s medication comprised: propranolol 10 mg twice daily, metformin 700 mg twice daily, dutasteride 0.5 mg daily, association of levodopa 100 mg and benserazide 25 mg twice daily, paroxetine 20 mg twice daily, and lorazepam 2.5 mg (at night). Rivastigmine (transdermic patch, 4.6 mg/24 hours) was started.

Five years later, the patient began complaining of auditory hallucinations, exclusively of musical choral nature, folk songs, nursery rhymes, well-known religious hymns, and even a popular football chant. These songs were present all the time, and were only to a certain extent attenuated by environmental noise. The patient initially thought the music was being played outside, but came to realize it was an odd phenomenon somehow generated in his brain. The patient was moderately upset and would like to be relieved, but did not attribute any frightening or persecutory meaning to the symptoms. There had been no change in levodopa, rivastigmine, antidepressant, or other medication before the musical hallucinations appeared. The psychiatry consultant prescribed low-dose risperidone (1 mg daily), but the patient became deeply sedated with urinary retention. Because of the auditory symptoms, the patient looked for the opinion of an otorhinolaryngology specialist, who diagnosed age-related hearing loss and prescribed bilateral hearing aids. Remarkably, after using the hearing aids, he did not hear the bothering songs any longer, only some tinnitus, described as a whistle. Even when he temporarily removed the hearing aids, the songs remained absent. However, at the same time, the patient began having visual hallucinations for the first time, namely he would see small spiders everywhere. Clozapine (6.25 mg daily) was initiated, with noticeable improvement of these visual hallucinations. Unfortunately, the general condition of the patient declined, he entered a nursing home, and died 1 year later.

## Discussion and conclusions

The present clinical case of a patient with DLB and age-related hearing loss illustrates the immediate shift from auditory hallucinations to visual hallucinations when bilateral hearing aids were used. To our knowledge, the immediate shift of hallucinations from one sensory modality to another sensory modality when perception is improved has not been previously described.

The patient now reported fulfilled initially the criteria for *possible* DLB, namely the presence of dementia plus a core clinical feature, Parkinsonism, and eventually reached the criteria for *probable* DLB, by developing visual hallucinations [10]. Visual hallucinations are typical of DLB, and represent a core feature of the disease, whereas hallucinations in other modalities are considered a supportive clinical feature [10]. Auditory hallucinations are also frequent in DLB, being described in about 30% of patients, and they may be verbal or nonverbal, but only rarely of musical type [11]. The presence of auditory hallucinations in patients with DLB is associated with hearing loss [12].

Musical hallucinations may improve when hearing loss is overcome by cochlear implantation. However, the procedure can paradoxically trigger or aggravate these hallucinations [13]. The placement of hearing aids in this patient with DLB led to the disappearance of the musical hallucinations, and only a whistle-like tinnitus persisted. At the same time, the visual hallucinations typical of DLB appeared. To our knowledge, the immediate shift of hallucinations from one sensory modality to another sensory modality when perception is improved has not been previously reported. From a theoretical point of view, the relevance of brain pathology to the genesis of hallucinations seems strengthened, in the sense that hallucination modality is directed to the most fragile sensory pathway. Recent electrophysiological studies have emphasized the role of uncoupling of the default mode network (DMN) from attention and control networks on the genesis of hallucinations and related symptoms in DLB [14] and other disorders, namely schizophrenia [15]. These attention and control networks also integrate and select peripheral signals to the somatosensory cortices [14]. It is thus understandable that the uncoupling of DMN would lead to disordered states of consciousness incorporating hallucinations from different sensory modalities. The present clinical case shows that these sensory modalities can shift by manipulation of peripheral sensory input.

A comment on the pharmacological treatment of musical hallucinations is worthwhile. Different drugs have been tried for the treatment of musical hallucinations, namely antipsychotic and antiepileptic drugs, with variable results, essentially on the basis of individual case reports [16]. The patient here described became deeply sedated and had urinary retention with a low-dose antipsychotic. The use of antipsychotics in patients with DLB should be avoided whenever possible, given the increased risk of a serious sensitivity reaction [10].

In conclusion, improving patients’ sensory deficits is advisable; however, the clinician should bear in mind and let the patient and caregiver know that improving one sensory modality to ameliorate hallucinations may sometimes paradoxically lead to hallucinations in a different sensory modality.

## Data Availability

The datasets supporting the conclusions of this article are available.
